# Efficient Gene Stacking in Rice Using the GA*A*NTRY System

**DOI:** 10.1186/s12284-021-00460-5

**Published:** 2021-02-06

**Authors:** Leyla T. Hathwaik, James Horstman, James G. Thomson, Roger Thilmony

**Affiliations:** United States Department of Agriculture-Agriculture Research Service, Western Regional Research Center, Crop Improvement and Genetics Research Unit, Albany, CA 94710 USA

**Keywords:** *Agrobacterium*, Gene stacking, Genetic engineering, *Oryza sativa*, Site-specific recombinase

## Abstract

Genetic engineering of rice provides a means for improving rice grain quality and yield, and the introduction and expression of multiple genes can produce new traits that would otherwise be difficult to obtain through conventional breeding. GA*A*NTRY (Gene Assembly in *Agrobacterium* by Nucleic acid Transfer using Recombinase technologY) was previously shown to be a precise and robust system to stably stack ten genes (28 kilobases (kb)) within an *Agrobacterium* virulence plasmid Transfer-DNA (T-DNA) and obtain high-quality Arabidopsis and potato transgenic events. To determine whether the GA*A*NTRY system can be used to engineer a monocotyledonous crop, two new T-DNA constructs, carrying five (16.9 kb) or eleven (37.4 kb) cargo sequences were assembled and transformed into rice. Characterization of 53 independent transgenic events demonstrated that more than 50% of the plants carried all of the desired cargo sequences and exhibited the introduced traits. Additionally, more than 18% of the lines were high-quality events containing a single copy of the introduced transgenes and were free of sequences from outside of the T-DNA. Therefore, GA*A*NTRY provides a simple, precise and versatile tool for transgene stacking in rice and potentially other cereal grain crops.

**Supplementary Information:**

The online version contains supplementary material available at 10.1186/s12284-021-00460-5.

## Background

Rice is one of the main staple food crops which feeds about half of the world’s population. It is estimated that by 2030, the world’s population is expected to reach 8 billion, and rice production must increase by 25% to meet the growing demand (Brar and Khush 2013). The exponential growth of omics (genomics, transcriptomic, proteomic and metabolomics) information offers substantial opportunities toward comprehending the mechanisms underlying rice grain quality and yield, furthermore, the integration of knowledge from this data with genetic engineering could potentially help achieve the needed sustainable increase in rice production.

Genetic engineering of rice has introduced several important traits such as herbicide tolerance, biotic/abiotic stress resistance and the improvement of nutritional value, as well as the quality of improvement of rice grains (Bao 2018; Biswal et al. 2017). The transgenic rice lines that have been most frequently developed typically express one or a few genes that confer traits of interest; nevertheless, modifying multiple traits simultaneously or introducing complex biosynthetic pathways will typically require the engineering of multiple genes. Therefore, rice biotechnology would significantly benefit from the application of a simple and versatile system for transgene assembly and efficient plant transformation.

Various different approaches have been utilized previously for the assembly of large plant transformation constructs, including the use of commercial cloning systems i.e. multisite Gateway, homing endonucleases, Gibson assembly, type IIS restriction enzymes, homologous recombination in yeast, as well as the Cre site-specific recombinase (Dafny-Yelin and Tzfira 2007; Ma et al. 2011; Weber et al. 2011; Untergasser et al. 2012; Zeevi et al. 2012; Buntru et al. 2013; Binder et al. 2014; Shih et al. 2016; Cermak et al. 2017; Zhang et al. 2017; Zhu et al. 2017). These approaches typically utilize either a binary vector plasmid or a binary bacterial artificial chromosome plasmid vector as the platform for the plant transformation construct. These efforts have produced large and complex multi-gene constructs, but in some cases have also been limited by construct instability problems in bacteria. Additionally, some were only shown to function in transient expression assays or in model plants, and/or were not able to efficiently generate stable transgenic plants exhibiting all the desired functional phenotypes. Site-specific recombinases have also been used to sequentially stack transgenes within a soybean target locus, and more recently within the rice genome (Li et al. 2010; Nandy et al. 2015; Pathak and Srivastava 2020), but these approaches require the generation of target lines with introduced sequences and perform stacking with multiple rounds of plant transformation. Clearly, plant biotechnology would significantly benefit from the application of a simple and versatile system that allows for the easy and stable assembly of large stacked constructs and their subsequent efficient and high-fidelity introduction into crop plants (Srivastava and Thomson 2016).

The GA*A*NTRY (Gene Assembly in *Agrobacterium* by Nucleic acid Transfer using Recombinase technology) system has proven to be an efficient method of gene assembly and plant transformation that generates high-quality transgenic Arabidopsis and potato plants (Collier et al. 2018; McCue et al. 2019). The GA*A*NTRY system is a simple to use, yet sophisticated method that allows the in vivo stacking of multiple transgenes within an *Agrobacterium* virulence plasmid Transfer-DNA (T-DNA). This system takes advantage of three unidirectional recombinases that mediate site-specific DNA recombination at their specific DNA recognition sites. The TP901 and A118 recombinases perform integration by recognition of their specific *attB* and *attP* recognition sites, while the ParA recombinase performs excision through its recognition of two directly oriented *MRS* recognition sites (Keravala et al. 2006; Thomson and Ow 2006; Thomson et al. 2009). The assembly of cargo sequences within the GA*A*NTRY’s T-DNA is an efficient, simple and modular process that only requires three types of components; 1) the modified GA*A*NTRY recipient *Agrobacterium* strain, 2) two cloning plasmids (B and P Donors) for the insertion of sequence(s) of interest and 3) two accessory plasmids (B and P Helpers) that carry an operon expressing two of the recombinases (for a detailed description of how the GA*A*NTRY assembly process functions, see Collier et al. 2018). Recently, the GA*A*NTRY system was demonstrated to efficiently assemble a 28.5 kilobase (kb) 10-stack T-DNA and produce high-quality genetically engineered Arabidopsis and potato plants (Collier et al. 2018; McCue et al. 2019).

To further investigate the use of GA*A*NTRY as a tool to genetically modify monocotyledonous crop plants, we sequentially stacked 5 or 11 cargo sequences to generate a 16.9 or 37.4 kb T-DNA respectively and produced genetically engineered rice. Transgenic events were evaluated phenotypically and genotypically to determine the completeness of T-DNA transfer and integration, as well as the quality of the events based of copy number and presence of sequences outside of the T-DNA’s left border (LB).

## Materials and Methods

### Construction of B and P Donors

Genes of interest (promoter/gene coding sequence/terminator) were assembled into either a B or P donor plasmid (Collier et al. 2018) using conventional restriction-ligation cloning methods. Ligated P/B donors were transformed and maintained in *E.coli* DH5α and plasmid DNA was extracted using a plasmid isolation kit according to manufacturer’s instructions (Zymo Research, CA). A summary of the P/B donors and their cargo sequences used to assemble the 5-stack and 11-stack T-DNAs is shown in Table 1. These plasmids and their complete annotated sequences are available on request.

**Table 1:** List of B and P Donor vectors used to assemble the 5-stack and 11-stack T-DNA

Stack #	Donor Vector		Phenotype	Cargo Sequence		
		Cargo Size (kb)		Promoter	CDS^**a**^	Terminator
**1**	B	3.4	hygromycin resistance	*RUBQ2*	*hptII*	*CaMV 35S*
**2**	P	2.1	*Renilla* luciferase activity	*CaMV 35S*	*Rluc*	*CaMV 35S*
**3**	B	2.2	enhancer blocking insulator	TBS (Transformation Booster Sequence)		
**4**	P	4.2	firefly luciferase activity	*OsCc1*	*Fluc*	*nos*
**5**	B	4.8	β-glucuronidase activity	*OsLP2*	*GUSPlus*	*nos*
**6**	P	3.1	glufosinate herbicide tolerance	*PvUbi1*	*bar*	*nos*
**7**	B	3.0	green florescence	*OsRoot6*	*eGFP*	*CaMV 35S*
**8**	P	2.2	enhancer blocking insulator	TBS (Transformation Booster Sequence)		
**9**	B	4.3	red florescence^b^	*OsPS2*	*tdTomato*^*ER*^	*nos/35S*
**10**	P	4.6	glyphosate herbicide tolerance	*OsGOS2*	*EPSPS*	*nos*
**11**	B	3.4	paromomycin resistance	*ZmUbi1*	*nptII*	*CaMV 35S*

^a^ CDS: gene coding sequence

^b^ The cargo 9 sequence was nonfunctional and failed to produce detectable red fluorescent pollen

### Multigene Stacking in the GA*A*NTRY Strain

**Fig. 1:**
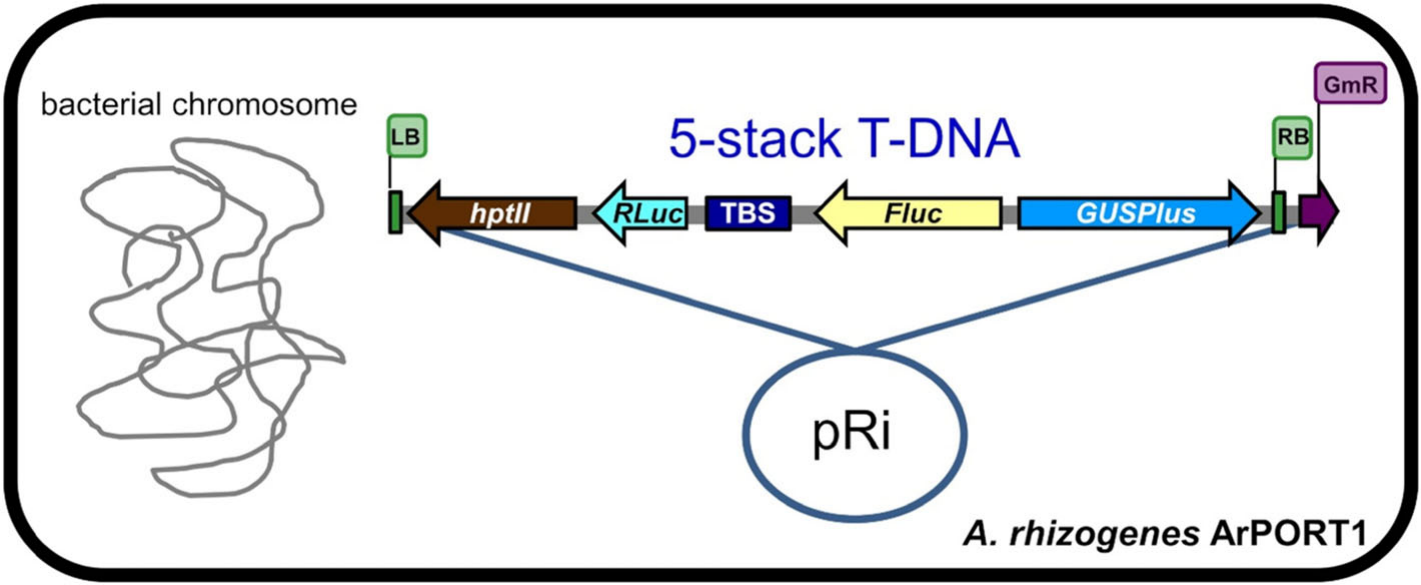
Composition of the 5-stack T -DNA. Diagram of an *Agrobacterium rhizogenes* virulence plasmid containing the 5-stack T-DNA. Cargo sequences, including the promoter, gene coding sequence and transcription terminator, are displayed as a colored arrow showing the orientation of transcription or a dark blue rectangle (TBS insulator). The T-DNA right border (RB) and left border (LB) locations are shown in green. Abbreviations are as follows: *hptII*, *hygromycin phosphotransferase 2, Rluc* (*Renilla luciferase)*, TBS: Transformation Booster Sequence, *Fluc* (*Firefly luciferase)* and *GUSPlus* (β-glucuronidase encoding reporter gene). The gentamicin bacterial resistance marker (*GmR*) is shown outside the RB region of the T-DNA, within the native *Agrobacterium* virulence plasmid (pRi)

**Fig. 2:**
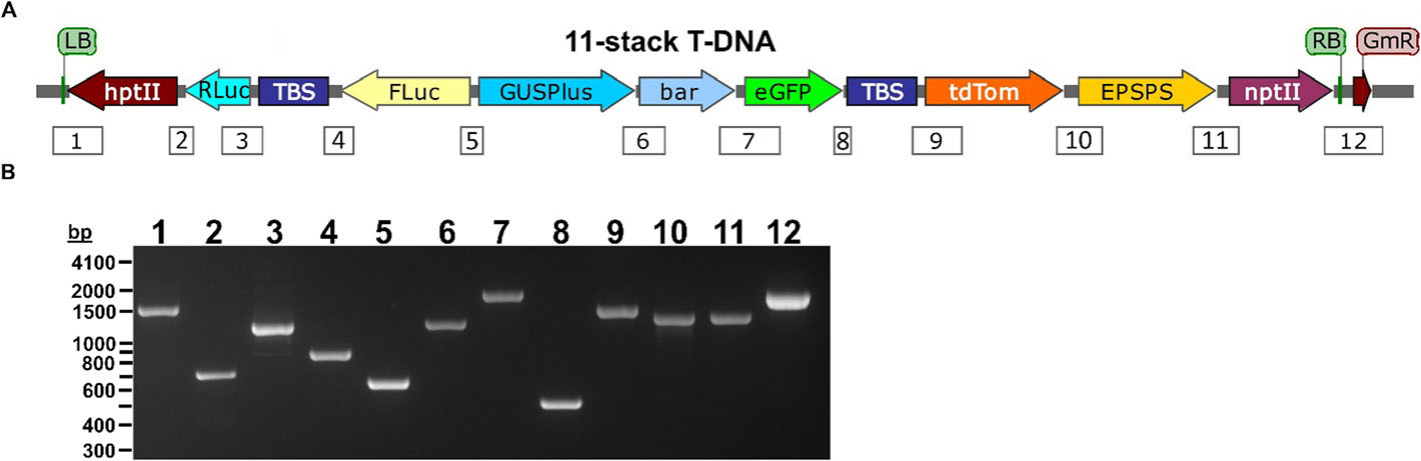
Composition of the 11-stack T -DNA and PCR validation of cargo sequences. **a** Diagram of the GA*A*NTRY 11-stack 37.4 kb T-DNA. See Fig. 1 legend for a description of the diagram. PCR products that bridge junctions between each cargo sequence are indicated by the numbered rectangles below the T-DNA. **b** Gel electrophoresis image of the PCR amplicons spanning each junction. Abbreviations are as follows: *hptII* (*hygromycin phosphotransferase 2*)*, Rluc* (*Renilla luciferase*), TBS (Transformation Booster Sequence), *Fluc* (*Firefly luciferase*), *GUSPlus* (β-glucuronidase encoding reporter gene), *bar* (*bialaphos resistance*), *eGFP* (*enhanced Green Fluorescent Protein*), *tdTom* (*tdTomato* red fluorescent protein), *EPSPS* (*5-enol-pyruvylshikimate-3-phospate synthase*), and *nptII* (*neomycin phosphotransferase 2)*

The GA*A*NTRY transgene stacking method (Collier et al. 2018) was used as previously described to stack cargo sequences in five or eleven steps within the GA*A*NTRY ArPORT1 strain (Fig. 1 and 2a). Briefly, GA*A*NTRY assembly is a simple process that employs iterative recombinase-mediated integration and excision reactions that toggle between the B Donor vectors (conferring bacterial gentamicin resistance) and P Donor vectors (conferring bacterial kanamycin resistance) each carrying the desired cargo. The B and P Donor vectors that were used to generate 5- and 11-stack GA*A*NTRY strains are summarized in Table 1. Note that the *nptII* expression cassette in the 11-stack construct was strategically added as the last stack to avoid any potential problems that would result from making the GA*A*NTRY strain constitutively resistant to kanamycin (if the *ZmUbi1*p-*nptII* expression cassette expressed in bacteria), which would make adding additional cargoes difficult with P Donor vectors carrying cargo sequences. The molecular characterization and validation of the strains generated from each stacking event involved picking three random colonies and performing genomic PCR reactions using gene specific primers that span the junctions between preexisting sequences and newly inserted cargo. The PCR amplification conditions used were an initial denaturation step of 5 min at 95 °C, followed by 35 cycles of 30 s at 95 °C, 30 s at 56 °C and 2 min at 68 °C, with a final extension at 68 °C for 5 min. The primers used for this analysis are shown in Supplemental Table 1.

### Examining the Stability of the GA*A*NTRY Stacked Cargo in *Agrobacterium*

To determine the stability of the stacked cargo within the GA*A*NTRY strain, the 5-stack and 11- stack strains were subcultured every 24 h over a period of 6 days in nonselective LB broth media and subsequently plated on nonselective LB agar. One hundred colonies were then picked and restreaked on LB media containing gentamicin. Then 10 colonies were randomly selected for cargo validation PCR using the primers shown in Supplemental Table 1.

### Generation of 5-Stack and 11-Stack Rice Plants

To generate transgenic rice (*Oryza sativa*) cv. Nipponbare plants, seed-derived embryogenic calli were co-cultured with *Agrobacterium rhizogenes* GA*A*NTRY strain carrying either the 5-stack or 11-stack T-DNA as previously described (Cook and Thilmony 2012). A single *Agrobacterium* -rice callus co-cultivation experiment was performed using the 5-stack strain to recover 16 independent events, while two separate co-cultivation experiments were performed with the GA*A*NTRY 11-stack strain to generate 37 independent events. Hygromycin antibiotic selection (40 mg/L) was used to identify transgenic rice callus. Regenerated shoots were grown in the greenhouse at 28 °C with 16 h of light in Sunshine Mix #1 soil medium (Sun Gro Horticulture, WA).

### Phenotypic Analysis of T_1_ Transgenic Rice Plants

#### Antibiotic and Herbicide Resistance

To determine antibiotic and herbicide resistance of GA*A*NTRY transgenic rice lines, selective germination and herbicide application assays were performed. Briefly, seeds were harvested from the T_0_ plants, dehulled, surface sterilized and subsequently transferred into 1 x MS medium containing the antibiotic and/or herbicide. The chemicals used were hygromycin (40 mg/L), paromomycin (125 mg/L), Finale® herbicide (22.5 μl/L, with a final concentration of 2.71 mg/L glufosinate), and Roundup® herbicide (12.2 μl/L, with a final concentration of 4.3 mg/L glyphosate) for seedling selection. The seeds on the selective media were placed in a growth chamber (Conviron, CA, USA) under 16 h of light at 28 °C. The seed germination and seedling growth was evaluated after 1 week. Hygromycin resistant seedlings were transferred to soil, grown in the greenhouse, and used to for scoring the other introduced phenotypes. In addition, Finale® herbicide (1:128 dilution, with a final concentration of 0.93 g/L glufosinate) was painted onto three-inch sections of leaves of one-month old plants with a herbicide dipped paint brush and scored for tolerance 7 days post application.

#### Dual Luciferase Reporter Assay

*Renilla* and firefly luciferase activities were measured in a single sample using two leaf disks (1 cm in diameter) using the dual-luciferase® reporter assay according to manufacturer’s instructions (Promega-E1910, WI, USA).

#### Microscopic Analysis

A Leica Microsystems-MZ16F stereomicroscope (Bannockburn, IL, USA) was used to examine green fluorescence in one-week-old roots and red fluorescence in pollen. The *eGFP* fluorescence visualization used an excitation wavelength of 488 nm with an emission filter 510–530 nm; for *tdTomato* fluorescence, an excitation wavelength of 558 nm with an emission filter 583–592 nm was used.

#### Histochemical GUSPlus Staining

To assay β-glucuronidase reporter enzyme activity, one-week old seedling leaves or whole seedlings were incubated with histochemical staining solution (0.1 M sodium phosphate pH 7.0, 0.5 mM potassium ferrocyanide, 1.5 g/L X-gluc (5-bromo-4-chloro-3-indolyl-β-D-glucuronic acid), and 0.5% *v*/*v* Triton X-10) for 18 h at 37 °C (Jefferson et al. 1987).

### Genotypic Analysis of T_1_ Transgenic Plants

Genomic rice DNA was extracted using the PureGene tissue DNA isolation kit (Qiagen, Valencia, CA, USA) from leaf tissue samples. Transgenic plants were analyzed by PCR using the same sequence-specific primers and amplification conditions that were used in the 5-stack and 11-stack GA*A*NTRY strain validation, as well as internal primers that anneal within individual cargo sequences (those marked with an asterisk, see Figs. 4 and 5) to verify the presence or absence of the cargo sequences (Supplemental Table 1).

### Measurement of Transgene Copy Number

The copy number of the *hptII*, *GUSPlus* and *nptII* cargo sequences were measured using droplet digital PCR (ddPCR) as previously described (Collier et al. 2017). Briefly, rice genomic DNA was isolated (Lassner et al. 1989) and quantified using a dsDNA quantification kit and a Qubit fluorometer according to the manufacturer’s instructions (Thermo Fisher Scientific, MA, USA). DNA was digested using an overnight incubation at 37 °C with the NcoI restriction enzyme (New England BioLabs, MA, USA). A total of 20 ng of digested DNA was used for each ddPCR reaction. Rice *OsUBC* (*Ubiquitin-Conjugating Enzyme E2*; LOC_Os02g42314) (Jain et al. 2006) gene was used the single copy reference gene. The reference gene probe was 5′ FAM (6-fluorescein)-labeled, and the transgene probes were 5′ HEX (hexachloro-fluorescein)-labeled. Primers and probes used for ddPCR analyses are shown in Supplemental Table 4.

### Screening for ‘Backbone’ Sequences from outside the T-DNA Left Border

Transgenic rice plants were analyzed for the presence of vector backbone sequence beyond the T-DNA left border using genomic PCR. The “LB” primers were designed to amplify a 1142 bp fragment that included sequences outside of the left border repeat (230 bp) and the 3′ end coding sequence of the *hptII* gene (755 bp) (within the T-DNA). Internal primers (PCR reaction 1*) which amplify a 435 bp fragment within the 3′ end coding sequence of the *hptII* gene were used as a positive control for the presence of cargo 1 and to be certain that negative PCR results were not caused by insufficient template or a faulty PCR amplification reaction. Additionally, the GA*A*NTRY strain carrying the 11-stack T-DNA was also used as a positive control for backbone sequence. See Supplemental Table 1 for primer sequences.

## Results

### Assembly of the 5 and 11-Stack GA*A*NTRY Strains

The GA*A*NTRY transgene stacking method was used to sequentially stack five or eleven sequences within the *Agrobacterium* virulence plasmid T-DNA (Fig. 1 and 2a). The selected cargo sequences (Table 1) were cloned into a P or B Donor plasmids, and multigene stacking within the GA*A*NTRY ArPORT1 strain was achieved by alternating the use of B and P Donor vectors and gentamicin and kanamycin selection to stack the genes of interest into the recipient GA*A*NTRY strain as previously described (Collier et al. 2018). The donors contained transcriptional units that were designed to confer functional phenotypes (i.e. antibiotic/herbicide resistance or reporter gene activity) and one cargo (which appears twice in the 11-stack T-DNA) was an enhancer-blocking insulator. The 5*-*stack GA*A*NTRY strain contains the *hptII*, *Renilla luciferase*, TBS insulator, firefly *luciferase* and *GUSPlus* cargoes, while the *bar, eGFP,* TBS insulator, *tdTomato, EPSPS* and *nptII* cargo sequences were added to the 5-stack to generate the 11-stack GA*A*NTRY strain (Table 1).

The Donors carrying each cargo sequence were iteratively stacked in either five or eleven independent steps to produce the 5-stack (16.9 kb) and 11-stack (37.4 kb) T-DNAs respectively (Fig. 1 and 2a). Although the 5- and 11-stack T-DNAs could have been constructed in fewer steps by designing donors that contained multiple cargos using Golden Gate-compatible and Gateway®-compatible auxiliary donor vectors (Collier et al. 2018), the 5 and 11 step pathways were chosen to demonstrate the repetitive and efficient nature of the GA*A*NTRY assembly system. Thorough molecular characterization of the assemblies was performed on three randomly selected *Agrobacterium* colonies via genomic PCR using gene-specific primers that span each of the recombination junctions between the cargo sequences (Fig. 2). All of the junctions in all tested colonies during each round of assembly were validated by genomic PCR screening, demonstrating the efficiency and reliability of the GA*A*NTRY-mediated cargo insertion process. Approximately equal number of colonies were obtained during each round of transformation from stack 1 to stack 11.

### Stability of the Stacked Cargo Sequences

To determine the stability of the stacked cargo within the ArPORT1 GA*A*NTRY strain pRi plasmid, the 5-stack and 11-stack strains were subcultured in nonselective LB broth medium every 24 h over a period of 6 days. Aliquots of the final culture were then plated on nonselective LB agar, and one hundred random colonies were screened using gentamicin selection. All one hundred colonies exhibited antibiotic resistance; ten of these were randomly selected for validation with genomic PCR and were confirmed to carry all expected cargo sequences. Representative PCR results are shown in Fig. 2b for an individual 11-stack clone.

### Generation and Characterization of 5-Stack and 11-Stack Transgenic Rice Plants

To examine the ability of the GA*A*NTRY strains to generate transgenic rice, Nipponbare wild-type embryogenic rice callus was co-cultured with either the 5 or 11-stack GA*A*NTRY strains. Transgenic calli was identified using hygromycin antibiotic selection. Sixteen and thirty-seven independent transgenic (T_0_) events were recovered using the GA*A*NTRY 5- and 11-stack strains respectively. The T_0_, T_1_ and T_2_ generations of each event were grown in the greenhouse to determine the functionality and stability of the introduced T-DNAs. Phenotypic and genotypic results indicate stable inheritance of the transgenes in each of the transgenic lines with no obvious differences observed in morphology or growth between the genetically engineered and wild-type Nipponbare rice plants.

**Fig. 3:**
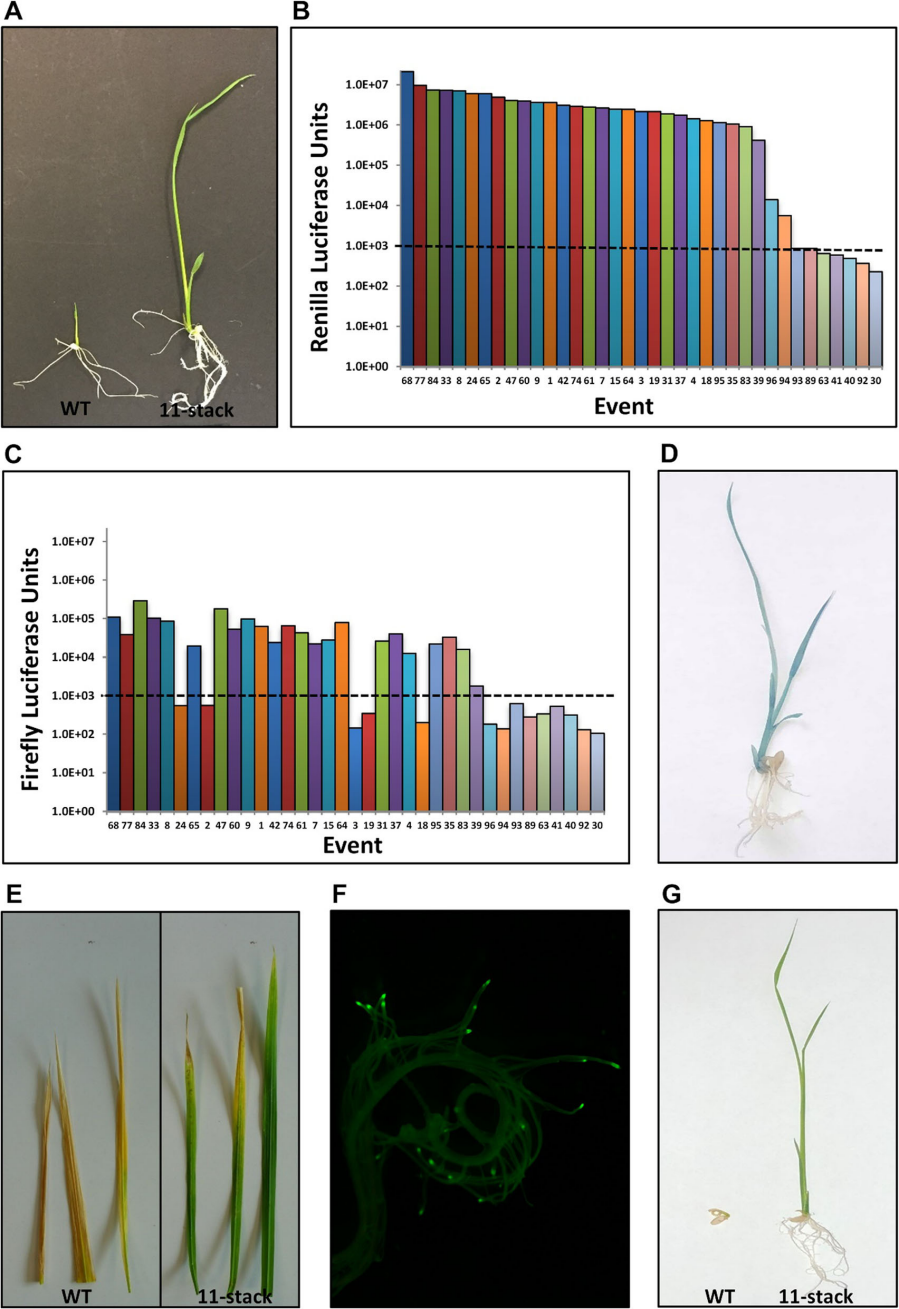
Representative phenotypes observed in the GA*A*NTRY 11-stack T_1_ rice plants. **a** Hygromycin sensitive wildtype Nipponbare (WT, left) and resistant (11-stack right) seedlings 7 days post germination. **b** Measured *Renilla* luciferase activity for 11-stack leaf samples. **c** Firefly luciferase activity for 11-stack leaf samples. The average background levels of activity detectable in wildtype leaf extracts is shown with the dashed line at 1.0 × 10^3^ units. **d** β-glucuronidase activity detected in green tissues and not in roots in a histochemically stained 7-day old 11-stack seedling. **e** Finale herbicide sensitivity in wildtype (left) and tolerance in 11-stack (right) leaves. **f** Observed green fluorescence localized in the root tips of a 11-stack rice plant. **g** The phenotypes of a wildtype (left) and 11-stack (right) seedlings germinated on media containing hygromycin and paromomycin antibiotics as well as glufosinate and glyphosate herbicides

**Fig. 4:**
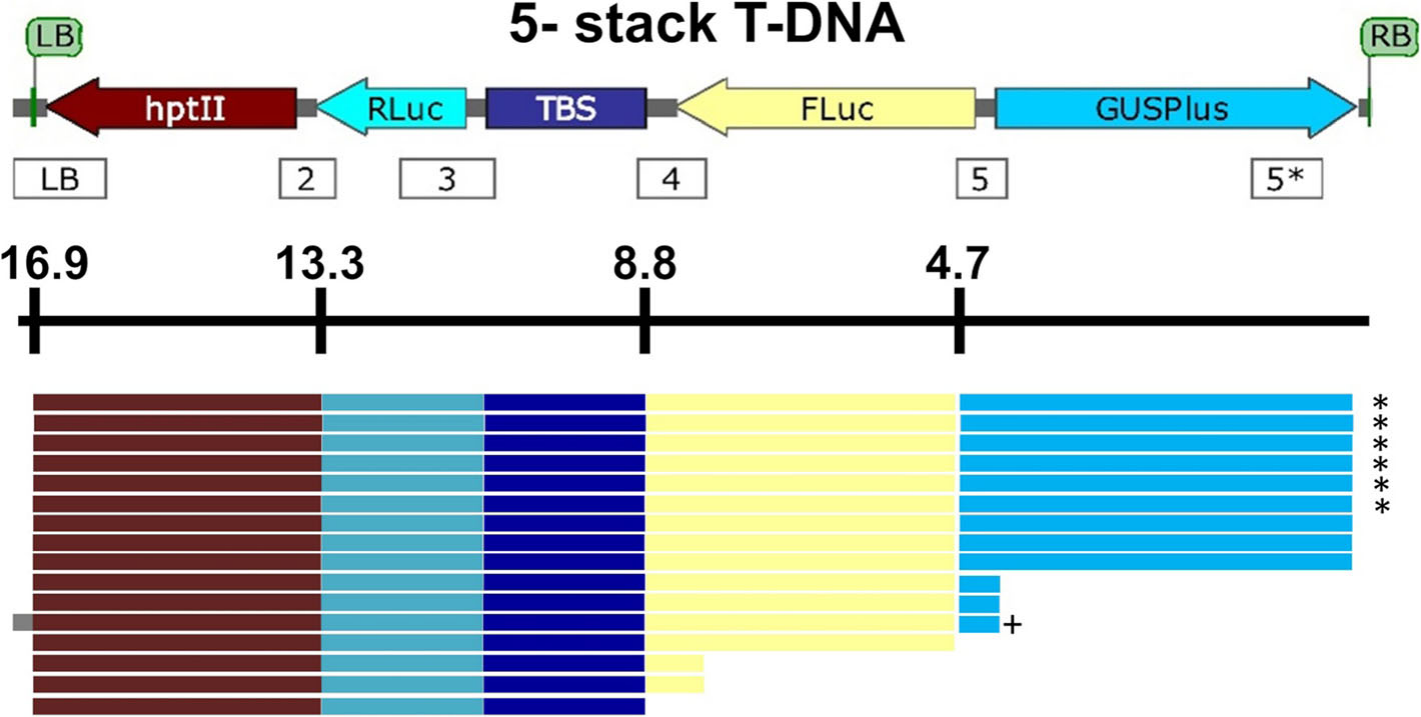
The GA*A*NTRY 5-stack T-DNA and summary of the phenotypes and genotypes of the independent events. The structure of the 16.9 kb 5-stack GA*A*NTRY T-DNA (top). PCR amplicons used to determine the presence of the T-DNA cargo sequences and the LB backbone sequence are indicated by the numbered rectangles below the T-DNA diagram. Amplicon 5* is fully within the GUSPlus cargo sequence, while the other amplicons span the junction between cargo sequences. The distance of each complete cargo sequence is from the right border (in kb) is shown below the diagram. Colored rectangles indicate the presence of the associated phenotype and/or genotype for each independent transgenic event. The asterisks (*) mark the events with a single copy of the *hptII* and *GUSPlus* transgenes. The plus sign (+) marks the event that contained sequence outside of the T-DNA beyond the left border

**Fig. 5:**
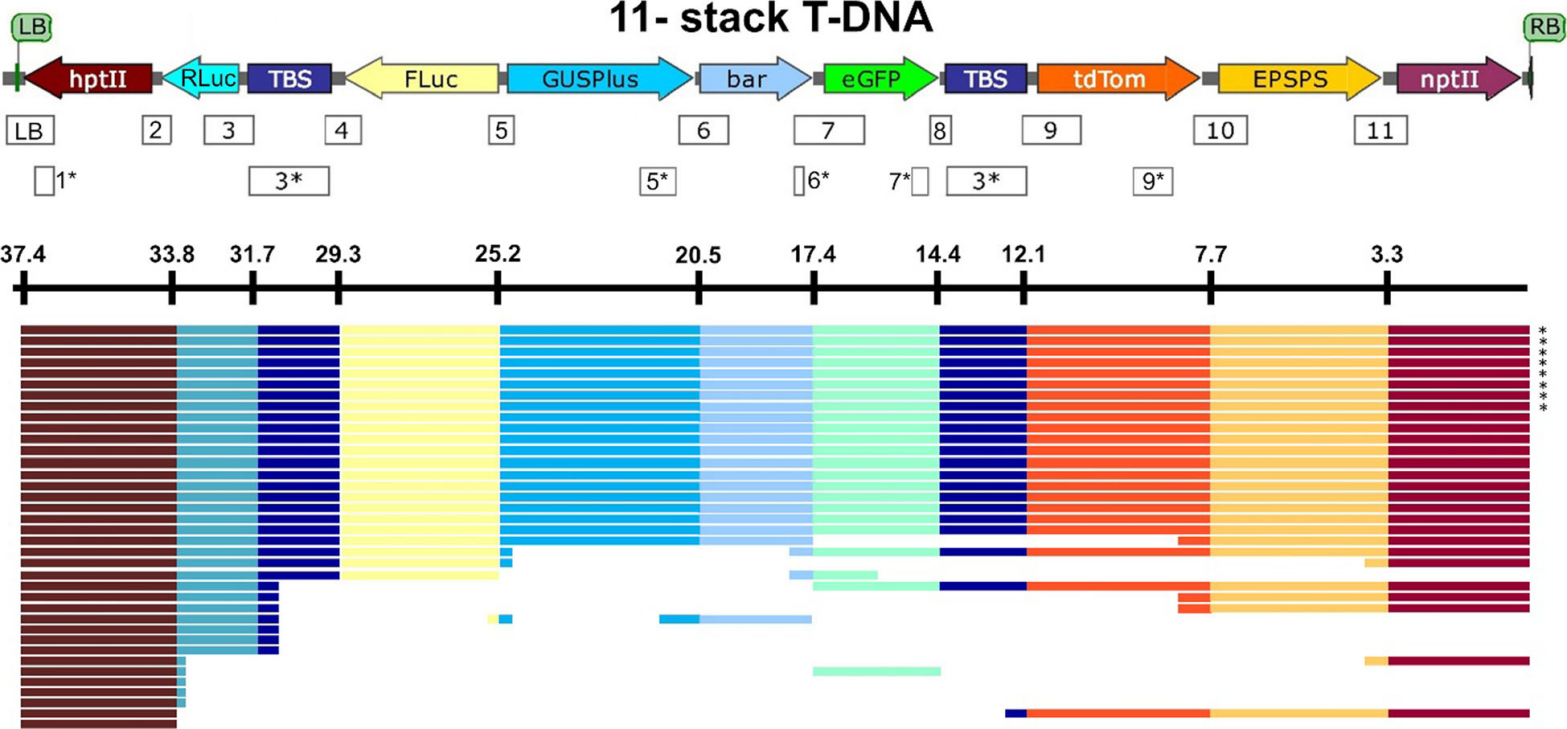
The GA*A*NTRY 11-stack T-DNA and a summary of the phenotypes and genotypes of the independent events. The GA*A*NTRY 37.4 kb 11-stack T-DNA (top). PCR amplicons used to determine the presence of the T-DNA cargo sequences and the LB backbone sequence are indicated by the numbered rectangles below the T-DNA diagram. Amplicons marked with an asterisk are within a single cargo sequence, while those without, span junctions between cargo sequences. The distance of each complete cargo sequence is from the right border (in kb) is shown below the T-DNA diagram. Colored rectangles indicate the presence of the associated phenotype and/or genotype for each independent transgenic event. The asterisks (*) mark the events with a single copy of the *hptII*, *GUSPlus* and *nptII* cargo sequences

The phenotypic expression of the introduced traits was analyzed in for each of the transgenic events. Cargo sequences 1 and 11 (Table 1) confer antibiotic resistance. The *hptII* gene is under the control of the rice *ubiquitin 2* promoter and *nptII* expression is conferred by the maize *ubiquitin 1* promoter; these genes confer resistance to hygromycin and paromomycin respectively (Collier et al. 2016). Germination assays were performed for T_1_ seed using antibiotic selection. As expected, all transgenic events produced progeny that were resistant to hygromycin, consistent with that antibiotic being used to select the original T_0_ transgenic events. A representative example of the hygromycin resistance observed is shown in Fig. 3a. The T_1_ seed from all 37 of the 11-stack events were germinated on paromomycin media and 73% had progeny that exhibited resistance (Supplemental Table 3; Fig. 3g). Cargo sequences 2 and 4 were expected to confer *Renilla* and firefly luciferase activity (Table 1). A dual-luciferase® activity assay was performed on leaf tissue extracts to assess which events had functional expression for each reporter gene. All 5-stack events exhibited *Renilla* luciferase activity and 81% had detectable firefly luciferase activity. Genomic PCR on the three events lacking activity confirmed that those plants also lacked at least a portion of the firefly luciferase expression cassette (Fig. 4, Supplemental Table 2). For the 11-stack events, 81% had detectable *Renilla* luciferase activity and 62% were positive for firefly luciferase activity (Fig. 3b-c, Supplemental Table 3). Similar to the 5-stack transgenic events, all of the 11-stack events that did not have luciferase activity also lacked that portion of the 11 stack T-DNA (Fig. 5, Supplemental Table 3).

Cargo sequences 3 and 8 were the Transformation Booster Sequence (TBS) from *Petunia* (Hily et al. 2009). This sequence is expected to block interactions between the CaMV 35S enhancer and nearby transgenes within the T-DNAs, thus maintaining the fidelity of their organ- or tissue-specific expression. The fifth cargo sequence was the *GUSPlus* expression cassette (Table 1) where expression of the reporter was controlled by the rice organ-specific *Leaf Panicle 2* promoter (Thilmony et al. 2009). For the 5-stack events, 56% exhibited the expected green tissue-specific *β-glucuronidase* activity via histochemical staining (Supplemental Table 2). Approximately 54% of the 11-stack events had detectable GUSPlus reporter activity only in leaves and other photosynthetic tissues (Fig. 3d, Supplemental Table 3). Most of the events lacking activity also lacked the *GUSPlus* portion of the T-DNA based on genomic PCR and ddPCR screening, although two of the 5-stack events and two of the 11-stack events carried at least a part of the *GUSPlus* construct sequence but failed to produce detectable histochemical staining (Figs. 4, 5, Supplemental Tables 2, 3).

The sixth cargo sequence in the 11-stack T-DNA was the *bar* gene under control of the constitutive switchgrass *Ubiquitin 1* promoter (*PvUbi1*) (Mann et al. 2011). Finale® herbicide was applied to individual leaves and 57% of the events showed tolerance (Fig. 3e). The events that were as sensitive to Finale® herbicide as the wildtype Nipponbare plants lacked the region of the T-DNA with the *bar* cargo sequence (Fig. 5, Supplemental Table 3). The seventh cargo sequence contained the *eGFP* (*enhanced Green Fluorescence Protein*) gene with its expression controlled by the rice *Root6* promoter which controls root-tip specific expression (Xing et al., unpublished). A total of 22 of the events (60%) exhibited root-tip specific green fluorescence and carried that portion of the 11-stack T-DNA (Fig. 3f, Supplemental Table 3). Cargo sequence 9 carried the red fluorescent *tdTomato* reporter gene under control of the rice *PS2* (also known as *OsLPS3*) pollen-specific promoter sequence (Oo et al. 2014). Even though 60% of the transgenic events carried the complete *tdTomato* expression cassette based on genomic PCR, none of the events exhibited detectable red fluorescence in pollen, or any other tissues (Fig. 5, Supplemental Table 3). Since the *tdTomato* transgene failed to generate a detectable phenotype in any of the 11-stack events, this sequence was considered a nonfunctional cargo sequence. The tenth cargo sequence was the *5-enol-pyruvylshikimate-3-phospate synthase* (*EPSPS)* gene under the control of the rice *GOS2* promoter (de Pater et al. 1992). Seedling germination assays determined that 68% of the events were tolerant to Round-up herbicide and genomic PCR screening confirmed that the 12 events that lacked herbicide tolerance, also lacked the *EPSPS* portion of the 11-stack T-DNA (Supplemental Table 3). Similarly, T_1_ seeds from each of the 11-stack events was germinated on paromomycin and 73% of the events exhibited antibiotic resistance and carried the 11th cargo sequence (Supplemental Table 3). Additionally, to confirm that the 11-stack plants can be both antibiotic and herbicide tolerant, a germination assay was performed with media that simultaneously contained hygromycin, paromomycin, glufosinate and glyphosate. An image of a sensitive wildtype Nipponbare seedling and a resistant/tolerant 11-stack seedling is shown in Fig. 3g.

Overall, for the GA*A*NTRY 5-stack events, 56% contained all four expected phenotypes (Fig. 4). Of the remaining events, 43% were lacking *β-glucuronidase* and 13% were missing firefly luciferase and *β-glucuronidase* activity. In the case of the GA*A*NTRY 11-stack events, 51% contained all eight functional phenotypes and appeared to have at least one copy of the entire 37 kb T-DNA. The remaining 49% of the events were missing one or more of the functional phenotypes and two or more of the cargo sequences (Supplemental Table 3). The phenotypes near the center of the T-DNA were the most frequently lacking in this set of events. The observed phenotypic and genotypic results from the 5- and 11-stack events remained stable in the T_2_ generation, indicating stable inheritance and expression of the integrated transgenes in each event.

### Quantification of Transgene Copy Number and Detection of Sequences from outside the T-DNA

**Fig. 6:**
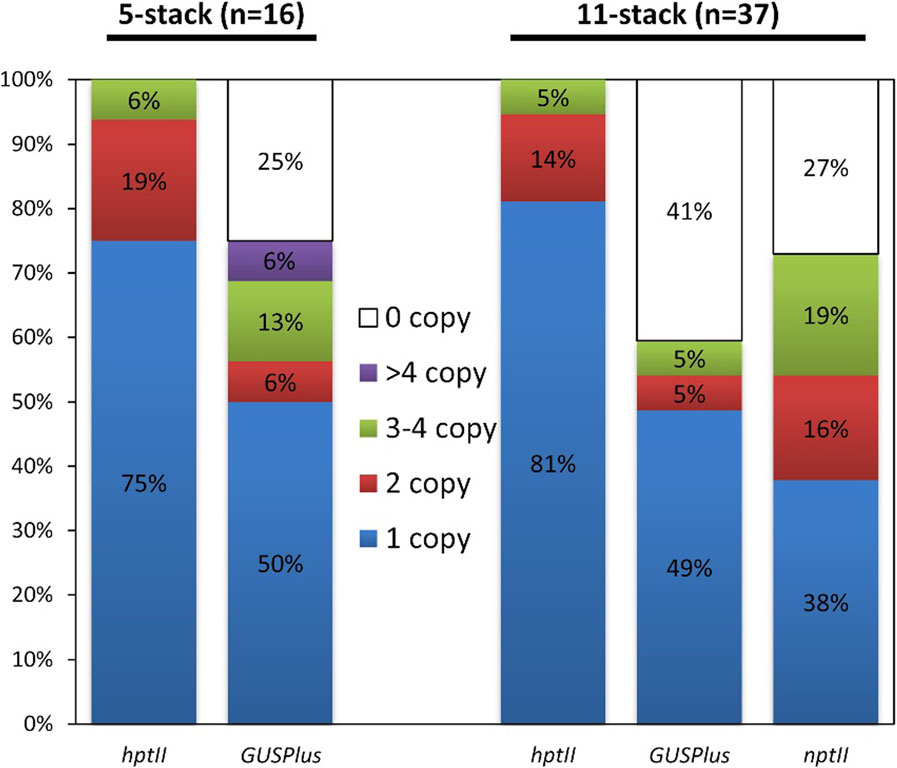
Transgene copy number in the GA*A*NTRY 5- and 11-stack events. The percent of total tested events with zero, one, two, three to four or more than four copies of the indicated transgenes (*hptII*, *GUSPlus* or *nptII*) is shown

The transgene copy number was measured using droplet digital PCR (ddPCR) in T_0_ transgenic 5-stack and 11-stack events. A summary of the results is shown in Fig. 6. For the 5-stack events, 38% were shown to likely have one copy of a complete T-DNA (i.e. they had 1 copy of both the *hptII* and *GUSPlus* transgenes and exhibited all four of the expected phenotypes) (Supplemental Table 2). For the GA*A*NTRY 11-stack events, 19% were high-quality/desirable lines carrying one copy of the *hptII*, *GUSPlus* and *nptII* cargo sequences, and exhibited all eight of the functional phenotypes (Figs. 3 and 5, Supplemental Table 3).

Overall, the copy number for the *hptII* transgene was quite low, with 75% and 81% of the 5-stack and 11-stack events having a single copy, respectively (Fig. 6, Supplemental Tables 2, 3). However, only 38% of the 11-stack events had a single copy of the *nptII* cargo sequence. Furthermore, ddPCR results confirmed the lack of portions of the T-DNA that correlated with the missing phenotypes. The transgenic 5-stack and 11-stack GA*A*NTRY rice plants were also screened by genomic PCR for the presence of introduced ‘backbone’ sequences. One 5-stack event (transgenic event 7; Fig. 4, Supplemental Table 2) had DNA from outside T-DNA left border, while the remaining 52 events were free of backbone sequences (Supplemental Tables 2 and 3).

## Discussion

Rapid progress in biotechnology and omics data availability has made it possible to enhance agronomic traits through genetic engineering. In contrast to monogenic traits to engineer herbicide or pest resistance, the genetic engineering of multiple traits or complex traits is more challenging and will involve multiple genes or whole biosynthetic or signaling pathways. Recently, the GA*A*NTRY system was demonstrated to be an effective technology that allowed the in vivo assembly and stable maintenance of a 10-stack 28.5 kb T-DNA within an *Agrobacterium* virulence plasmid and to successfully produce high quality transgenic events in both *Arabidopsis thaliana* and potato plants (Collier et al. 2018; McCue et al. 2019). In this report, the GA*A*NTRY system was tested to assess its capacity to generate transgenic rice plants using new 5-stack and 11-stack assemblies carrying cargo sequences which are more suitable for functioning in monocots. The GA*A*NTRY system was again shown to effectively assemble and stably maintain large multigene T-DNA stacks, as the 11-stack T-DNA was 30% larger than the previously published 10-stack, totaling 37.4 kb. The assembly process was also demonstrated to be a simple and reliable process that can allow numerous iterative rounds of the integration of cargo into a GA*A*NTRY *Agrobacterium* strain T-DNA. It was also shown that not only is the assembly process reliable, but the assembled cargo is stably maintained in the GA*A*NTRY strain, even when grown for days without antibiotic selection. Although individual transgene cassettes were assembled into the T-DNA in as many as eleven rounds of site-specific recombination and selection, it is entirely possible that through the use of Golden Gate-compatible and Gateway®-compatible auxiliary donor vectors (Collier et al. 2018), large multigene assemblies of ten genes or more could be constructed in as few as two or three rounds of GA*A*NTRY assembly, making this a very rapid and efficient process.

The use of the 5-stack and 11-stack GA*A*NTRY strains allowed us to generate genetically engineered rice expressing multiple traits. The utilization of both constitutive and organ-specific promoters to control the expression of the introduced transgenes, in combination with the TBS enhancer-blocking insulator, demonstrated that continuous and spatio-temporal/organ-specific expression could be achieved with a single large T-DNA construct. The 11-stack T-DNA contained constitutively expressed genes for hygromycin and paromomycin resistance, glufosinate and glyphosate herbicide tolerance, and two forms of luminescence conferred by the *Renilla* and firefly luciferases. In addition, the engineered lines had green tissue organ-specific *β-glucuronidase* activity and exhibited detectable green fluorescence only within the root tips. Surprisingly, use of a rice pollen specific promoter fused to the *tdTomato* reporter gene failed to generate detectable fluorescence in pollen (as expected) or any other tissues. An explanation for this unexpected result is not obvious, since the entire expression cassette was sequence confirmed within the 11-stack T-DNA and the *tdTomato* transgene and *PS2* pollen-specific promoter has been confirmed functional in Nipponbare rice in other transformation constructs (data not shown).

Analysis of the 5-stack and 11-stack transgenic plant populations (T_0_, T_1_ and T_2_) that were recovered using hygromycin selection showed that more than half contained the entire stacked T-DNA (based on phenotype and genotype assays). The other events, based on genomic PCR analyses, were typically lacking portions of the T-DNA which explained their failure to produce the expected phenotypes. Since selection of transgenic plants was conducted using only hygromycin selection, it is not surprising that most of the missing T-DNA regions were located within the middle or RB proximal regions of the GA*A*NTRY T-DNAs. This suggests that in the future, if researchers want to more frequently recover events carrying an entire large stacked construct, localizing selection markers near both the left and right borders of the T-DNA and utilizing two selection agents within the transformation and regeneration process will likely enrich for the recovery of events carrying the entire desired construct. However, even with hygromycin selection only, 19% of the 11-stack events were high-quality desirable lines that appeared to carry 1 complete copy of the 37.4 kb T-DNA, were backbone-free and expressed all eight of the detectable introduced phenotypes.

## Conclusion

To date, GA*A*NTRY strains containing multigene T-DNA stacks have been successful at producing high-quality transgenic events in the model plant Arabidopsis (Collier et al. 2018) the *Solanaceous* tuber crop of potato (McCue et al. 2019) and now the staple cereal crop of rice. The system has been shown to generate low copy, backbone-free, functional events at a substantial frequency with large T-DNA constructs. In this study, six (38%) and seven (19%) 5-stack and 11-stack GA*A*NTRY lines appeared to have one complete T-DNA since they have a single copy of *hptII*, *GUSPlus* and *nptII* (11-stack only) transgenes and expressed all the introduced functional traits. Additionally, out of all 53 transgenic rice events, only a single 5-stack line tested positive for sequences outside of the T-DNA construct, showing that the GA*A*NTRY system most frequently allows the transfer and integration of only the T-DNA sequences. The results presented show that the GA*A*NTRY system provides a simple and reliable method to generate large T-DNA constructs that can be efficiently used to produce high-quality transgenic rice.

## Supplementary Information

**Additional file 1: Supplemental Table 1.** Primers used for genomic PCR reactions to validate the insertion of cargo sequences within the GAANTRY 5-stack and 11-stack strains and the molecular characterization of transgenic rice events. Related to Figs. 2, 4 and 5.

**Additional file 2: Supplemental Table 2.** Summary of the phenotype and genotype data for the 5-stack transgenic events. Related to Figs. 4 and 6.

**Additional file 3: Supplemental Table 3.** Summary of the phenotype and genotype data for the 11-stack transgenic events. Related to Figs. 3, 5 and 6.

**Additional file 4: Supplemental Table 4.** Primers and probes used in ddPCR analysis. Related to Fig. 6.

## Data Availability

Most of the data generated or analyzed during this study are included in this published article and its supplementary information files. Additional data or materials not included in published article are available from the corresponding author on reasonable request.

## Abbreviations

*attB*: *attachment Bacteria* recognition site
*attP*: *attachment Phage* recognition site
*bar*: *bialaphos resistance*
CaMV 35S: Cauliflower Mosaic Virus 35S
CDS: Gene coding sequence
DNA: Deoxyribonucleic Acid
ddPCR: Droplet digital Polymerase Chain Reaction
*eGFP*: *Enhanced Green Fluorescent Protein*
*EPSPS*: *5-enol-pyruvylshikimate-3-phospate synthase*
*Fluc*: Firefly *luciferase*
FAM: 6-fluorescein
GA*A*NTRY: Gene Assembly in *Agrobacterium* by Nucleic acid Transfer using Recombinase technology
GmR: Gentamicin Resistance
HEX: Hexachloro-fluorescein
*GUSPlus*: *β-glucuronidase*
*hptII*: *Hygromycin phosphotransferase 2,* LB: T-DNA Left Border
*MRS*: *Multimer Resolution Site*
*nptII*: *Neomycin phosphotransferase 2*
*nos*: *Nopaline synthase*
*OsCc1*: *Oryza sativa Cytochrome c1*
*OsLP2*: *Oryza sativa Leaf Panicle 2*
*OsPS2*: *Oryza sativa Pollen Specific 2*
*OsRoot6*: *Oryza sativa Root 6*
*OsUBC*: *Ubiquitin-Conjugating Enzyme E2*
PCR: Polymerase Chain Reaction
pRi: *Agrobacterium rhizogenes* virulence plasmid
*PvUbi1*: *Panicum virgatum Ubiquitin 1*
RB: T-DNA Right Border
*Rluc*: *Renilla luciferase*
*RUBQ2*: *Rice Ubiquitin 2*
TBS: Transformation Booster Sequence
T-DNA: Transfer-DNA
*tdTom*: *tdTomato* endoplasmic reticulum localized red fluorescent protein
X-gluc: 5-bromo-4-chloro-3-indolyl-β-D-glucuronic acid
*ZmUbi1*: *Zea mays Ubiquitin 1*
